# Removal of Hg^2+^ with Polypyrrole-Functionalized Fe_3_O_4_/Kaolin: Synthesis, Performance and Optimization with Response Surface Methodology

**DOI:** 10.3390/nano10071370

**Published:** 2020-07-14

**Authors:** Zhenfeng Lin, Ziwei Pan, Yuhao Zhao, Lin Qian, Jingtao Shen, Kai Xia, Yongfu Guo, Zan Qu

**Affiliations:** 1 Center for Separation and Purification Materials &Technologies, Suzhou University of Science and Technology, Suzhou 215011, China; Linzf@sjhb.cn (Z.L.); 1813022033@post.usts.edu.cn (Z.P.); 1713022014@post.usts.edu.cn (Y.Z.); 1911022009@post.usts.edu.cn (L.Q.); 1913022011@post.usts.edu.cn (J.S.); 1713022009@post.usts.edu.cn (K.X.); 2 Suzhou Sujing Environmental Engineering Co., Ltd., Suzhou 215122, China; 3 Jiangsu Collaborative Innovation Center of Technology and Material of Water Treatment, Suzhou 215009, China; 4 Jiangsu Provincial Key Laboratory of Environmental Science and Engineering, Suzhou University of Science and Technology, Suzhou 215009, China; 5 School of Environmental Science and Engineering, Shanghai Jiao Tong University, Shanghai 200240, China; quzan@sjtu.edu.cn

**Keywords:** magnetic material, polypyrrole, kaolin, response surface methodology, mercury

## Abstract

PPy-Fe_3_O_4_/Kaolin was prepared with polypyrrole functionalized magnetic Kaolin by a simple, green, and low cost method to improve the agglomeration and low adsorption capacity of Kaolin. PPy-Fe_3_O_4_/Kaolin was employed to remove Hg^2+^ and the results were characterized by various methods. Relevant factors, including solution pH, dosage of adsorbent, concentration (*C*_0_), and temperature (*T*), were optimized by Response Surface Methodology (RSM) and Central Composite Designs (CCD). The optimal results show that the importance for adsorption factors is pH > *T* > *C*_0_ > dosage, and the optimal adsorption conditions of PPy-Fe_3_O_4_/Kaolin are pH = 7.2, *T* = 315 K, *C*_0_ = 50 mg/L, dosage of 0.05 g/L, and the capacity is 317.1 mg/g. The adsorption process conforms to the pseudo-second-order and Langmuir models. Dubinin–Radushkevich model shows that adsorption process is spontaneous and endothermic. Moreover, the adsorption of mercury by PPy-Fe_3_O_4_/Kaolin was achieved mainly through electrostatic attraction, pore diffusion, and chelation between amino functional groups and Hg^2+^. PPy-Fe_3_O_4_/Kaolin has excellent reproducibility, dispersity, and chemical stability, and it is easy to be separated from solution through an external magnetic field. The experiments show that PPy-Fe_3_O_4_/Kaolin is an efficient and economical adsorbent towards mercury.

## 1. Introduction

With the enhancement of global industrialization level, the phenomenon of industrial wastewater pollution is more serious. The water pollution derived from industrial wastewater comes from an increasingly extensive range of sources, such as metallurgy, mining, printing and dyeing, chemistry, machining, and so on. Especially, various heavy metals in industrial wastewater, including mercury (Hg), copper (Cu), lead (Pb), chromium (Cr), and their compounds, have the characteristics of enrichment in the living things and biological toxicity. These environmental pollutions can lead to a serious harm to the human digestive system, urinary system, and nervous system [1,2], etc. Among them, mercury is very hazardous due to its high toxicity, extensive distribution, strong bioaccumulation, and easy flow [3,4]. Thus, it is especially emergent to remove mercury ions from water body quickly and efficiently.

Current methods for removing heavy metals and mercury ions from water body mainly include physicochemical and biological technologies [5,6,7,8,9]. However, these technologies face many challenges of high cost, low efficiency, cumbersome operation, and even unsafe preparation process [10,11]. Up to present, the adsorption technology has made great progress and it has unique advantages in the treatment of heavy metals and mercury [8,12]. And, adsorption technology has been largely applied to remove heavy metals and mercury in wastewater [6,13]. Common adsorbents in adsorption technology have been widely studied and applied, and include activated carbon [14], CNT [15], cellulose [16], titanium dioxide [17], silica gel [18], coffee waste [13,19], alkynyl carbon [20], clay [21], chitosan [22], functional groups [5,23,24], etc.

As a kind of common clay minerals, Kaolin has been largely applied in wastewater treatment, as a result of its stable constitution, distinctive crystal configuration, high specific surface areas, and abundant reserves [25]. Its surface has a uniform negative charge originated from the isomorphous Al^3+^ in Si^4+^ in silicon dioxide layer. Therefore, Kaolin is a potential sorbent in water treatment [26], like cationic dyes [25,27], antibiotics [28], aromatic compounds [29], and heavy metals [30]. However, when compared with the other traditional adsorbents, Kaolin has a serious agglomeration phenomenon and low adsorption capacity [31], which greatly limits its application in water treatment. Moreover, the research focus of Kaolin is to remove dyes and antibiotics from water. Additionally, there is little report about the elimination of mercury in water due to the poor adsorption capacity of Kaolin.

To overcome above shortcomings, various composite Kaolin have been researched by modification with carbon materials [32], organic compounds, acids [33], and polymers [34,35], etc. However, the above modification method is cumbersome, complicated and time consuming. Even some toxic, harmful, or hazardous substances (toluene, o-xylene or acetone, etc.) are employed as reaction media during the process of preparing composite materials.

As a kind of common polymer, polypyrrole (PPy) has a series of advantages, including easy to large scale, outstanding stability, and cheap cost [36]. During the preparation process of PPy, many functional groups may be introduced into the supports, which can produce strong adsorption for some cations by electrostatic attraction [36]. According to our previous research, the combination between PPy and magnetic graphene oxide (MGO) or CoFe_2_O_4_@SiO_2_ with a core–shell structure can achieve a high adsorption capacity for divalent mercury ions (Hg^2+^) in water [37].

Therefore, in the current study, a novel composite clay nanomaterial of PPy-Fe_3_O_4_/Kaolin was successfully prepared by PPy functionalized magnetic Kaolin and used to remove divalent mercury ions from water by a simple, green, and low cost method. Some effect factors, including solution pH, temperature, coexisting ions, and additive amount of pyrrole, were researched. Meanwhile, to investigate the optimal experimental parameters, a Central Composite Design (CCD) matrix founded on the RSM method was also employed. In addition, the recyclability and stability of the materials, as well as adsorption mechanism, were all investigated.

## 2. Materials and Experimental Methods

### 2.1. Chemicals and Materials

Ferric chloride (FeCl_3_·6H_2_O), anhydrous sodium acetate (CH_3_COONa), Kaolin, polyethylene glycol, pyrrole (Py), sodium dodecyl benzene sulfonate (SDBS), ethylene glycol (EG), and ammonia water (20–25 wt.%) were all obtained from Aladdin Reagent (Shanghai, China).

### 2.2. Synthesis of PPy-Fe_3_O_4_/Kaolin

**Figure 1:**
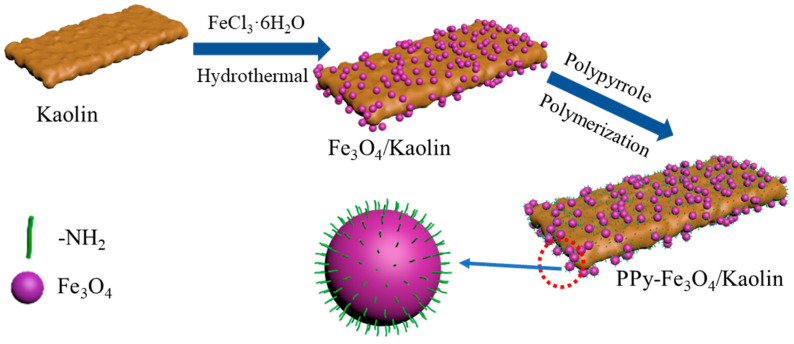
Prepared process of PPy-Fe_3_O_4_/Kaolin.

Figure 1 displays the prepared process of PPy-Fe_3_O_4_/Kaolin. 6.0 g of FeCl_3_·6H_2_O and 5.0 g CH_3_COONa were dissolved in 200 mL EG solution and ultrasonically dispersed for 0.5 h. Subsequently, 3.0 g Kaolin was added and ultrasonically dispersed for 2 h. Afterwards, 0.8 g polyethylene glycol was added and mechanically stirred for 0.5 h. After that, above solution was moved in a reaction kettle to be heated at 473 K for 14 h. The reaction kettle is a circular Teflon-lined autoclave with a volume of 100 mL. Magnetic Kaolin (Fe_3_O_4_/Kaolin) was separated with an exterior magnet of sintered NdFeB and then washed with ethanol and pure water after separation. The obtained production Fe_3_O_4_/Kaolin was desiccated at 333 K in order to produce a magnetic Kaolin powder.

Subsequently, 0.15 g magnetic Kaolin powder and 0.025 g SDBS were added in 100 mL distilled water and then ultrasonically dispersed for 0.5 h with continuously agitation. 0.15 mL Py monomer was added in the above solution containing magnetic Kaolin. 10 mL distilled water containing 3.0 g FeCl_3_·6H_2_O was added dropwise after being ultrasonically dispersed for 10 min. The mixed solution was continued to be stirred for 6 h. The obtained sample was separated and then rinsed with distilled water. Finally, the as-prepared PPy-Fe_3_O_4_/Kaolin was desiccated at 333 K and reserved in brown glass bottle. The above ultrasonic dispersion was carried out via an ultrasonicator with power of 240 W, voltage of 220 V, and frequency of 40 KHz.

### 2.3. Batch experiments

In the current experiment, a stock solution containing 1000 mg/L Hg^2+^ was provided for the experiment with a protective solution. The specific procedure can be found in our previous research [38]. The effects of pH, temperature, concentration of mercury solution, contact time, and additive quantity of pyrrole on the property of the PPy-Fe_3_O_4_/Kaolin were all investigated. The solution with metal specie and sorbent was mechanically stirred while using a vertical downward stirring shaft with length of 5 cm at 250 rpm during the process of adsorption.

After adsorption, the solution samples were firstly filtered through a syringe filter (0.45 μm) and, subsequently, determined by a cold atomic absorption adsorption spectrophotometry to calculate the equilibrium adsorption capacity *q*_e_ (mg/g) based on initial concentration (*C*_0_, mg/L) and residual concentration (*C*_e_, mg/L) in solution samples after adsorption equilibrium.

#### 2.3.1. Effect of pH

Solution pH containing Hg^2+^ was adjusted into a desired value within 2 to 8 with 0.1 M HCl and 0.1 M NaOH solutions. The experiment was performed under the conditions of mercury solution volume (*V*) of 100 mL, *C*_0_ of 40.0 mg/L, adsorbent dosage of 0.05 g/L, temperature (*T*) of 298 K, and time (*t*) of 7 h.

#### 2.3.2. Effect of Additive Amount of Pyrrole

Based on the preliminary experiments of adsorbent dosage, the dosage of 0.05 g/L was selected as the optimal preliminary parameter. The result can be found in Figure S1. 0.05 g/L adsorbents with different additive amounts of pyrrole (0.05, 0.10, 0.15, 0.20, 0.25, 0.30, and 0.35 mL) were added to a conical bottle with 100 mL Hg^2+^ sample (*C*_0_ = 40 mg/L, pH = 7). The above conical bottle was vibrated with a frequency of 250 rpm at 298 K for 7 h.

#### 2.3.3. Kinetic Experiments

0.05 g/L adsorbents was put in 100 mL mercury solutions (*C*_0_ = 40 mg/L, pH = 7) with a frequency of 250 rpm at 298 K. Afterwards, 0.005 mL solution was taken to analyze at various contact times of 1, 3, 5, 10, 15, 25, 40, 60, 90, 120, 180, 240, 300, 360, 420, and 480 min.

#### 2.3.4. Isothermal and Thermodynamic Experiments

Isothermal and thermodynamic experiments were carried out with 100 mL Hg^2+^ solutions containing various contents (20, 30, 40, 50, and 60 mg/L) and 0.05 g/L adsorbent under the conditions of frequency of 250 rpm, contact time of 7 h, pH of 7, and different reaction temperatures (298, 308, and 318 K).

#### 2.3.5. Recycle and Regeneration Experiment

Except the dosage of adsorbent, the other test conditions are the same as the effect of additive amount of pyrrole. 0.05 g/L of adsorbent was employed as the first adsorption test. After that, the used adsorbent was collected, and then soaked in 100 mL of HCl solution (0.1 M) for 7 h, which is used as the regenerant. The mixture is continuously stirred for 2 h under acidic conditions. Afterwards, the adsorbent was collected with an exterior magnet of sintered NdFeB with a diameter of 30 mm and thickness of 5 mm, which was placed at the bottom of the beaker containing a magnetic kaolin solution. Subsequently, the adsorbent was rinsed three times with distilled water for subsequent use. The above operation was repeated five times.

## 3. Results and Discussion

### 3.1. Characterizations

**Figure 2:**
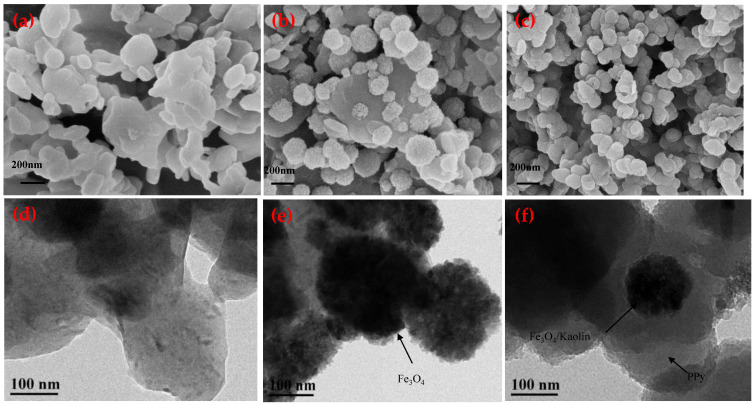
SEM images of (**a**) Kaolin, Magnification: 50,000×, (**b**) Fe_3_O_4_/Kaolin Magnification: 50,000× and (**c**) PPy-Fe_3_O_4_/Kaolin, Magnification: 20,000×; TEM images of (**d**) Kaolin, (**e**) Fe_3_O_4_/Kaolin, and (**f**) PPy-Fe_3_O_4_/Kaolin.

SEM and TEM technologies were used to observe the morphology and structure (Figure 2). As shown in Figure 2a, the shape of Kaolin is flaky with a smooth surface. A large number of spherical particles with a diameter of 50 to 100 nm are attached to the surface of the Kaolin (Figure 2b), which indicates that the successful synthesis of Fe_3_O_4_ particles on the surface of Kaolin. The PPy decorated Fe_3_O_4_/Kaolin shows denser and stacked spherical particles (Figure 2c). The spherical morphology may lead to great specific surface area (BET value) and excellent adsorption.

Figure 2d shows that Kaolin is flaky and has a smooth edge. Figure 2e,f show that large black and spherical particles were formed after Kaolin was combined with Fe_3_O_4_, and PPy materials were coated outside of the Fe_3_O_4_/Kaolin.

Energy-dispersive spectrometer (EDS) was employed to analyze the type and content of constituent elements in the materials, in conjunction with SEM and TEM analysis, as shown in Figure S2. From Figure S2, the material of PPy-Fe_3_O_4_/Kaolin is mainly made of oxygen (O), nitrogen (N), silicon (Si), Aluminum (Al), and iron (Fe), and the elements in the synthetic material are uniformly distributed, which is beneficial to the uniform adsorption of Hg^2+^. Moreover, Figure S2g shows the distribution of weight ratio of main elements in the material of PPy-Fe_3_O_4_/Kaolin. Among them, the contents of Fe and N are 17.0% and 17.5%, respectively, indicating the successful synthesis of PPy-Fe_3_O_4_/Kaolin.

**Figure 3:**
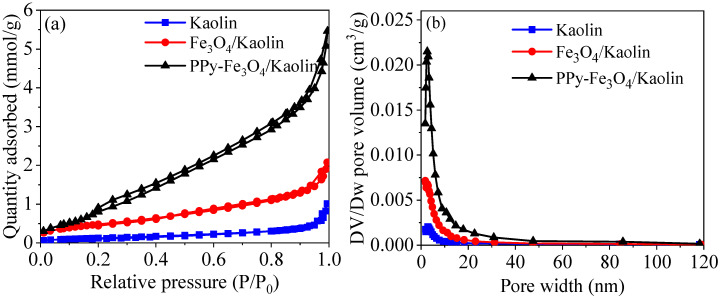
(**a**) N_2_ adsorption-desorption plot and (**b**) pore distribution plot.

N_2_ adsorption-desorption plots with Barret–Joyner–Halenda (BJH) method was used in order to determine the porous property, and the results are displayed in Figure 3. From Figure 3a, the isotherm of three materials belong to type-IV and hysteresis loop can be classified as type H3, which indicates a mesoporous property (0–20 nm). As shown in Figure 3b, the pore diameters of three materials of Kaolin, Fe_3_O_4_/Kaolin, and PPy-Fe_3_O_4_/Kaolin are 11.53, 6.65, and 8.31 nm based on the calculation of BJH method, respectively. In addition, the values of specific surface area (BET value) of Kaolin are significantly increased after modification, and the BET value of PPy-Fe_3_O_4_/Kaolin increases to 84.19 m^2^/g from 10.30 m^2^/g of Kaolin. Table S1 lists the results of adsorption-desorption of N_2_.

Despite the pore diameter of PPy-Fe_3_O_4_/Kaolin becoming smaller, but the total pore volume becomes larger, as shown in Table S1. The results of enlarged BET and total pore volume are beneficial for enhancing the adsorption performance of PPy-Fe_3_O_4_/Kaolin.

**Figure 4:**
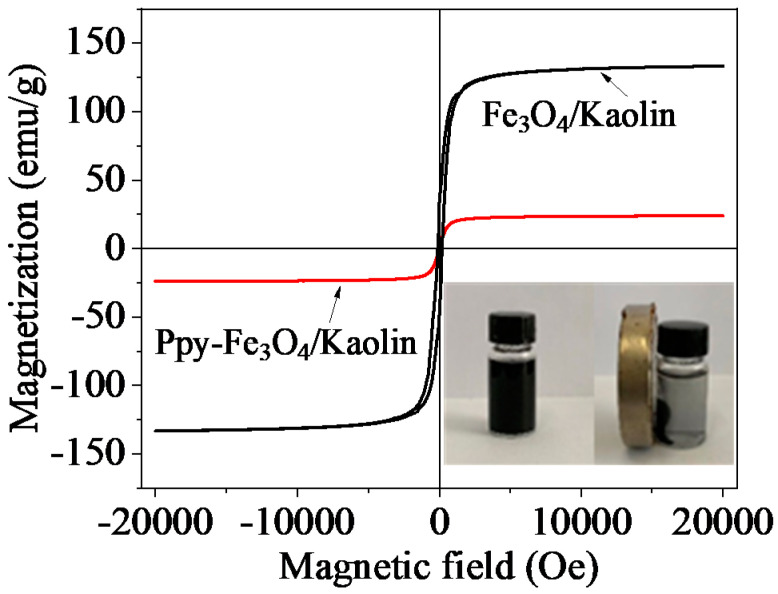
Magnetic hysteresis loops of Fe_3_O_4_/Kaolin and PPy-Fe_3_O_4_/Kaolin (The inserted picture is the effect of magnetic separation with an outer magnet).

Figure 4 displays the magnetic hysteresis loop of Fe_3_O_4_/Kaolin and PPy-Fe_3_O_4_/Kaolin. The saturation magnetization of Fe_3_O_4_/Kaolin and PPy-Fe_3_O_4_/Kaolin are 133.2 and 23.9 emu/g, respectively, indicating excellent magnetic properties, as shown in the insert of Figure 4. As polypyrrole is coated onto Fe_3_O_4_/Kaolin, the magnetic strength of PPy-Fe_3_O_4_/Kaolin is not as good as Fe_3_O_4_/Kaolin, but it is still easy to be separated quickly from the solution. The coercivity and remanence of PPy-Fe_3_O_4_/Kaolin are 202.5 Oe and 33.2 emu/g, respectively.

**Figure 5:**
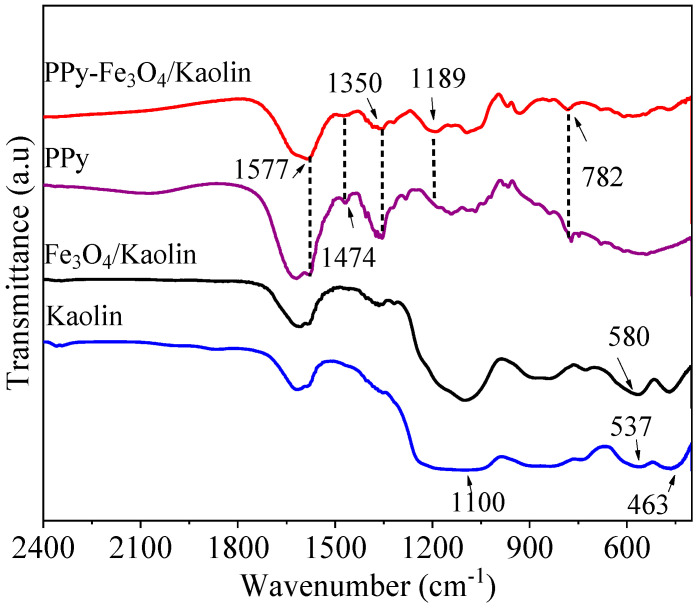
FT-IR of the as-prepared three materials.

Figure 5 displays the Fourier transform infrared spectrophotometer (FT-IR) results of Kaolin, Fe_3_O_4_/Kaolin, and PPy-Fe_3_O_4_/Kaolin. In the spectrum of Kaolin, the peak of around 1100 cm^−1^ should be ascribed as Si–O–Si vibration [39]. The wide peaks of 537 and 463 cm^−1^ belong to Si–O–Al skeletal vibration [40]. In the Fe_3_O_4_/Kaolin spectrum, the peak around 580 cm^−1^ can be ascribed as the vibration of Fe_3_O_4_ [24]. Moreover, the characteristic peaks of Kaolin have not been changed, indicating that iron oxide was successfully prepared on the surface of Kaolin. In PPy-Fe_3_O_4_/Kaolin, the peak at 1577 cm^−1^ represents existence of PPy [36,41], based on the C=C stretching. The peaks of 1189 and 1350 cm^−1^ are C–H vibration and C–N stretching [36], respectively. The peak of 782 cm^−1^ is N–H stretching vibration [42]. The stretching of C-N in the pyrrole ring is fixed at 1474 cm^−1^.

In addition, the peak intensities of Kaolin and Fe_3_O_4_/Kaolin are weakened due to the cover of PPy on the surface of Fe_3_O_4_/Kaolin. Based on the above results, it can be easily deduced that PPy is successfully coated outside of Fe_3_O_4_/Kaolin, which is consistent with the data of X-ray Diffraction analysis (XRD), as shown in Figure S3.

**Figure 6:**
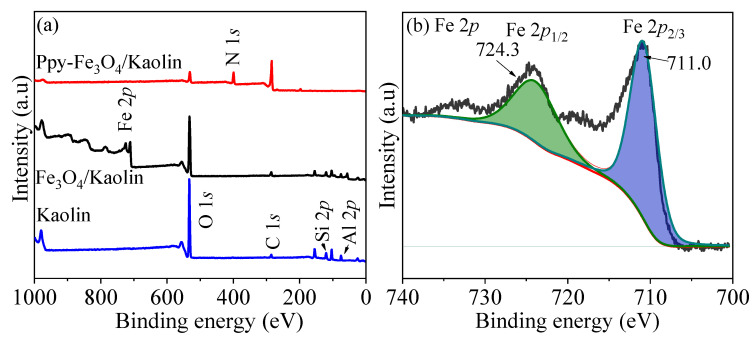

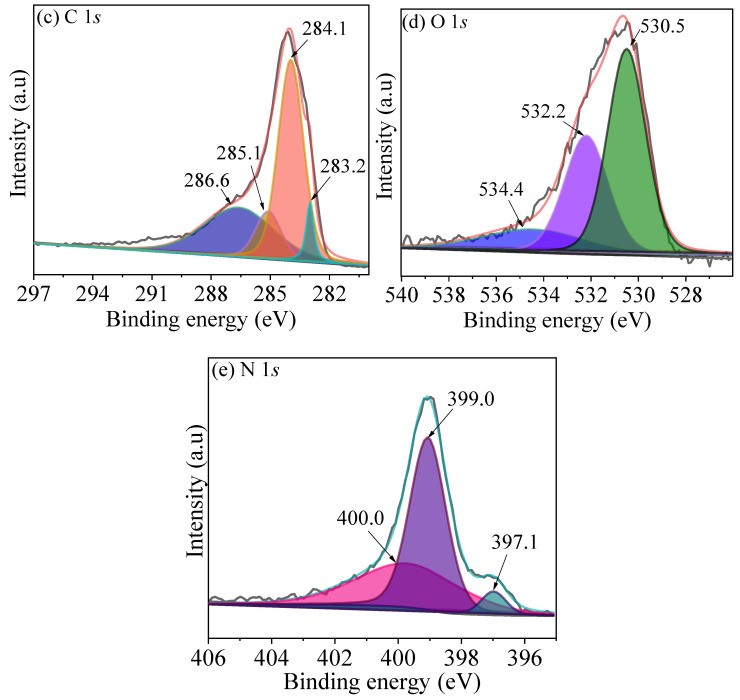
XPS spectra of (**a**) Kaolin, Fe_3_O_4_/Kaolin, PPy-Fe_3_O_4_/Kaolin, (**b**) Fe 2*p*, (**c**) C 1*s*, (**d**) O 1*s,* and (**e**) N 1*s*.

Figure 6 shows the XPS results of Kaolin, Fe_3_O_4_/Kaolin, and PPy-Fe_3_O_4_/Kaolin. From Figure 6a, four peaks in the X-ray photoelectron spectroscopy (XPS) of Kaolin at 285.20, 532.58, 74.90, and 103.35 eV can be found, which correspond to C 1*s*, O 1*s*, Al 2*p*, and Si 2*p*, respectively [40]. XPS spectrum of Fe_3_O_4_/Kaolin has a vibration at around 710 eV, which is ascribed to Fe 2*p*. In Figure 6b, there are two peaks of 711.0 and 724.3 eV represent Fe 2*p*_1/2_ and Fe 2*p*_2/3_, respectively. It indicates that iron element is loaded onto Kaolin. Moreover, it can be known from Table S2 that the Fe 2*p* mass ratio is about 2.6 wt.%. In the spectrum of PPy-Fe_3_O_4_/Kaolin, the peak of N 1*s* appears at 399.07 eV and the intensities of the other peaks are relatively weakened. It may be caused by polypyrrole coating on the outside of Fe_3_O_4_/Kaolin, which weakens the influence of other elements.

In Figure 6c, the C 1*s* peak is dominated by carbon at 284.1 eV and its mass ratio is 63.9 wt.%. The binding energy (B.E.) of 285.1 eV is ascribed to sp3 hybridized carbon. The binding energy of 286.6 eV is attributed to C=N/C-O [43,44]. Moreover, in Figure 6d, there are three peaks at 530.5, 532.2, and 534.4 eV, corresponding to the oxygen in carbonyl group, the oxygen atoms in hydroxyl ions and water [23], respectively. In Table S2, the proportion of O 1*s* is 12.8 wt.%.

In addition, it can be concluded that the mass percentage of N element is about 16.9 wt.%, which consists of -N= (13.5 wt.%), -NH- (61.8 wt.%) and N^+^ (24.7 wt.%), corresponding to 397.1 eV, 399.0 eV and 400.0 eV, as shown in Figure 6e, respectively. The above data indicate that the as-prepared PPy-Fe_3_O_4_/Kaolin has rich amino groups, and PPy was successfully synthesized on the surface of Fe_3_O_4_/Kaolin, which is consistent with the data of FT-IR and XRD.

**Figure 7:**
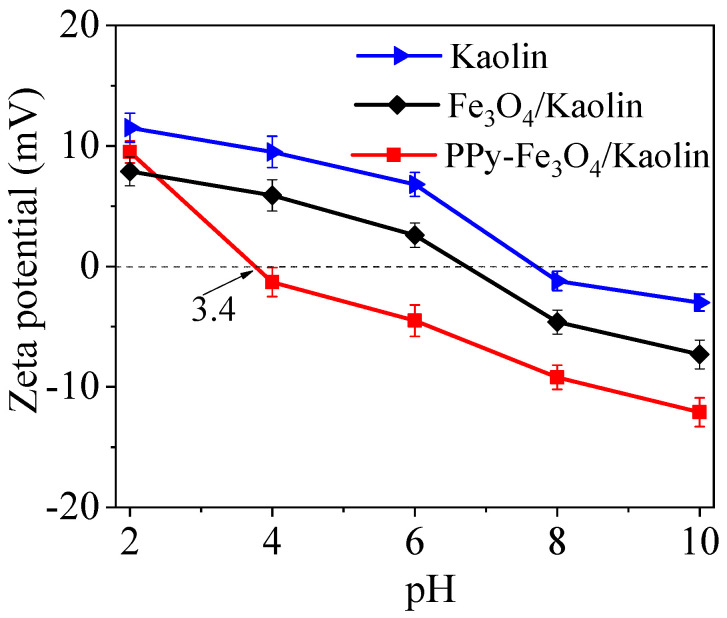
Zeta potential of Kaolin, Fe_3_O_4_/Kaolin and PPy-Fe_3_O_4_/Kaolin.

Zeta potentiometer was used to detect the surface potential of the Kaolin, Fe_3_O_4_/Kaolin, and PPy-Fe_3_O_4_/Kaolin, as shown in Figure 7.

It can be seen that the points of zero charge (pH_z_) of Kaolin is 7.7, and the pH_z_ of Fe_3_O_4_/Kaolin is 6.7. It is noteworthy that the pH_z_ of Kaolin is quite different from that reported in some literature, which is probably caused by the analysis methods, test method, or test instrument, etc. After being grafted with PPy, the pH_z_ of PPy-Fe_3_O_4_/Kaolin reduces to 3.4. The zeta potential of PPy-Fe_3_O_4_/Kaolin is −6.9 mV at pH of 7, which is much lower than those of Kaolin (2.8 mV) and Fe_3_O_4_/Kaolin (−1.1 mV). The relatively low zeta potential of PPy-Fe_3_O_4_/Kaolin solution is beneficial for reducing the agglomeration of the adsorbent and improving the removal of Hg^2+^.

### 3.2. Adsorption Performance Test

#### 3.2.1. Effect of pH

**Figure 8:**
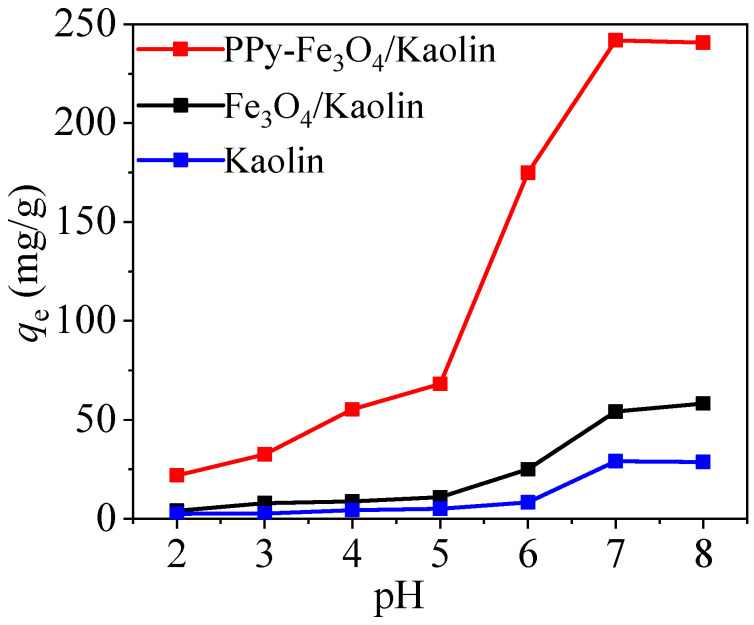
Influence of pH with Kaolin, Fe_3_O_4_/Kaolin, and PPy-Fe_3_O_4_/Kaolin.

Figure 8 shows the pH effects on the adsorption performance of Kaolin, Fe_3_O_4_/Kaolin, and PPy-Fe_3_O_4_/Kaolin. As pH ascends, all of the adsorption capacities of the three materials are increasing. The materials of Kaolin and Fe_3_O_4_/Kaolin have relatively low adsorption capacities at pH values of 2–8. However, the adsorption performance of Fe_3_O_4_/Kaolin has a significant enhancement and it reaches 241.8 mg/g at pH of 7. The result indicates a successful modification of Kaolin with PPy.

#### 3.2.2. Effect of Additive Amount of Pyrrole

**Figure 9:**
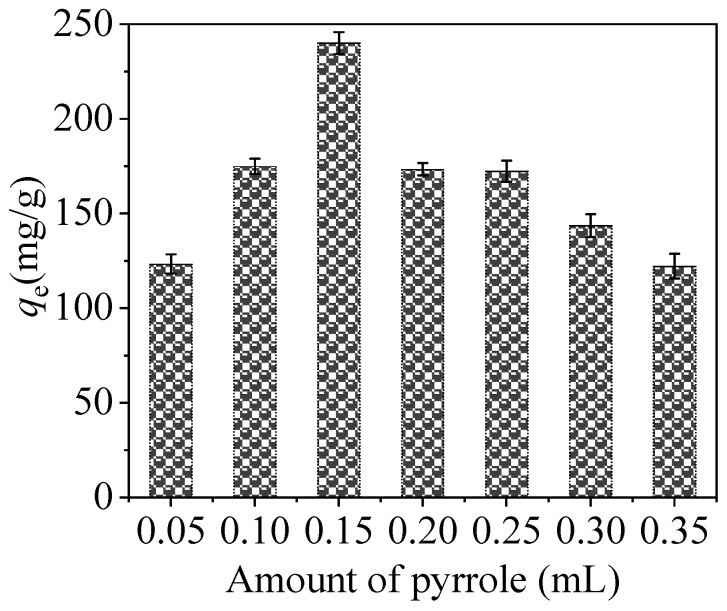
Effect of additive amount of Py on the performance of PPy-Fe_3_O_4_/Kaolin.

The investigation was implemented to research whether the additive amount of pyrrole added has an influence on the removal of Hg^2+^. From Figure 9, as an additive amount of pyrrole increases, the adsorption capacity of PPy-Fe_3_O_4_/Kaolin also ascends, and the maximal capacity of 241.8 mg/g occurs in the case of 0.15 mL pyrrole. When the additive amount of pyrrole is more than 0.15 mL, the adsorption performance of PPy-Fe_3_O_4_/Kaolin is obviously reduced. The reason may be that too much dosage of pyrrole will make polypyrrole gather together and reduce the number of effective active sites of PPy-Fe_3_O_4_/Kaolin. Therefore, the pyrrole amount of 0.15 mL was chosen for the experiments.

### 3.3. Optimization of Experimental Condition by RSM

In order to investigate the optimal experimental condition, the CCD matrix depended on the RSM method was generated using design expert 11.0. Four variables of pH (A), T (B), *C*_0_ (C) and dosage (D) were involved and run 30 runs, which were devised with five coded value points (−2, −1, 0, +1, +2), 18 design points in six axes, and six duplicates at the center clock. *q*_e_ is the response variable. Table S3 displays the corresponding data of running sequence experiments.

ANOVA is employed to investigate the significance and relationship between responses and variables [45]. The models that represent the relationship between responses and variables are expressed in quadratic form, as follows.

(1) $$\begin{array}{cc} q_{e} & =287.31+39.24A+11.82B+19.46C+4.68D+9.48\mathrm{AB}+10.35\mathrm{AC}\\ & -6.27\mathrm{AD}-4.39\mathrm{BC}-5.64\mathrm{BD}-0.3919\mathrm{CD}-{25.47A}^{2}-{4.22B}^{2}-{2.54C}^{2}-{14.31D}^{2} \end{array}$$

(2) $$\begin{array}{ll} q_{e} & =-1817.56+319.39\times \mathrm{pH}+10.04\times T+0.01\times C_{0}+23270.32\times Dosage\\ & +1.90\times \mathrm{pH}\times T+1.03\times \mathrm{pH}\times C_{0}-627.00\times \mathrm{pH}\times Dosage-0.09\times T\times C_{0}\\ & -112.86\times T\times Dosage-3.92\times C_{0}\times Dosage-25.50\times {\mathrm{pH}}^{2}-0.17\times T^{2}\\ & -0.03\times C_{0}{}^{2}-14306.40\times Dosage^{2} \end{array}$$

The fitting results with four models of Linear, Interaction (2FI), Quadratic, and Cubic modes are displayed in Table 1. It can be seen that the Quadratic mode has maximal coefficients of determination (*R*^2^ = 0.999) and minimum discrepancy of 0.008 between *R*_adj_^2^ and *R*_pre_^2^. Thus, the Quadratic mode can be the most suitable model for describing mercury adsorption by PPy-Fe_3_O_4_/Kaolin among the above four modes.

The antagonism and synergy among pH (A), T (B), *C*_0_ (C), dosage (D), and *q*_e_ are represented with plus and minus symbols. In the Quadratic mode, the values of A (pH), B (*T*), C (*C*_0_), and D (Dosage) reflect the systemic effect among them. From Table S4, Quadratic mode has a high F-value of 69260.83, implying Quadratic mode is significant. Additionally, from the four F-values, it can be known that the synergy significance among the variables is: A (pH) > B (*T*) > C (*C*_0_) > D (Dosage). The *p*-values of A (pH), B (*T*), C(*C*_0_), D(Dosage), AB, AC, AD, BC, BD, A^2^, B^2^, and C^2^ closes to zero, which proves that these terms have a remarkable influence on the *q*_e_ value. The *p*-values of CD and Lack of Fit are 0.704 and 0.069, respectively, indicating an insignificant effect.

**Figure 10:**
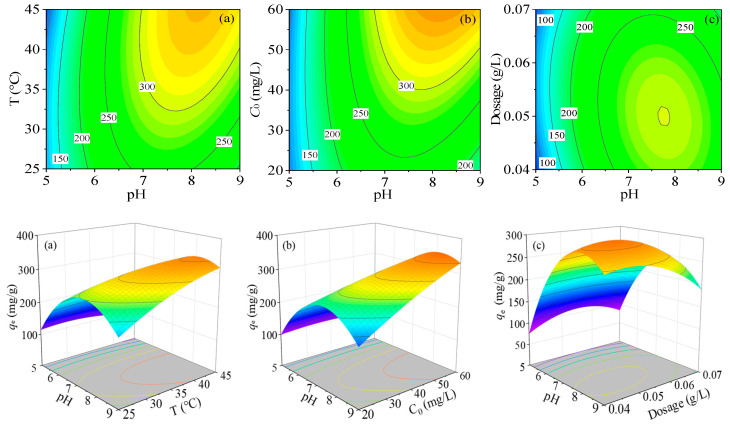

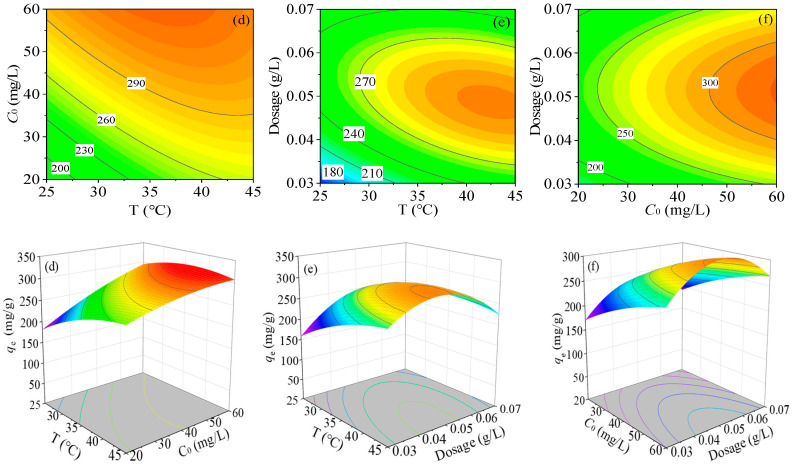
Three-dimensional (3D) relationship between variables and *q*_e_. (pH and temperature (**a**); pH and *C*_0_ (**b**); pH and dosage (**c**); temperature and *C*_0_ (**d**); temperature and dosage (**e**) and *C*_0_ and dosage (**f**)).

Figure 10a shows the effect of pH and temperature on the performance of the PPy-Fe_3_O_4_/Kaolin. It can be known from Figure 10a that the material of PPy-Fe_3_O_4_/Kaolin has better adsorption performance under the conditions of pH = 6–9 and temperature of 298 K–318 K. Moreover, as the pH values and temperature are increasing, the adsorption performance of PPy-Fe_3_O_4_/Kaolin gets bigger. The minimal *q*_e_ generates at a pH < 6 and low temperatures. The results show that increasing temperature is favorable to the adsorption of Hg^2+^ and the adsorption process is probably an endothermic reaction.

Combined with the data of zeta potentials that are shown in Figure 7 and the effect of pH in Figure 8, it can be known that the surface of the material is electronegative and possesses a lot of negative charges at high pH value (more than pH_z_). These negative charges can provide more equipotential points to adsorb mercury ions through electrostatic attraction. Meanwhile, the high F-value of AB and very small mean that the term of pH (A) and temperature (B) is significant when combined with the data in Table S4.

A three-dimensional plot of the combined effect of pH (A) and *C*_0_ (C) on capacity, which is performed at a temperature at 308 K and the dosage of PPy-Fe_3_O_4_/Kaolin at 0.05 g/L, as shown in Figure 10b. With the increasing pH and *C*_0_, the *q*_e_ of PPy-Fe_3_O_4_/Kaolin also ascends. When the pH and *C*_0_ are in the ranges of 7 to 8.5 and 40 to 60 mg/L, respectively, the adsorption capacity of PPy-Fe_3_O_4_/Kaolin is the largest. The reason is probably that only when the mercury ion reaches a specific concentration (40 to 55 mg/L) can the material’s adsorption capacity reach a saturated state under certain conditions (*T* = 308 K, *dosage* = 0.05 g/L). Also, in the Table S4, the term of pH and *C*_0_ is significant, due to a high F-value (633) and very low *p*-value (<0.0001).

Figure 10c displays the interaction between pH (A) and dosage (D) with PPy-Fe_3_O_4_/Kaolin (*T* = 308 K, *C*_0_ = 40 mg/L). From Figure 10c, the adsorption performance changes little as the raising dosage under a certain pH value. The fitted data indicates that the dosage may be insignificant during the process of adsorption, and the effect factor is mainly the variation of pH. The possible reason is that the adsorption performance of the material is fixed for a specific adsorbent under certain conditions. When the pH is set at 7.5–8.5 and the dosage at 0.045 g/L–0.055 g/L, the maximal adsorption capacity occurs. Furthermore, the fitted data of term AD (F-value = 252, *p*-value < 0.0001) also show that the term AD is less important.

Figure 10d shows the combined effect of temperature (B) and *C*_0_ (C) on the value of *q*_e_. The experiment was performed under the conditions of pH = 7 and *dosage* = 0.05 g/L. It can be known from the experimental results that when temperature is between 308 K and 313 K and *C*_0_ in the range of 40 to 45 mg/L, the PPy-Fe_3_O_4_/Kaolin has very strong adsorption capacity for Hg^2+^. This may be since the entire adsorption process is an endothermic reaction, as analyzed above. It can also be derived from the F-value (172.49) and *p*-value (<0.0001) of BC in Table S4 that the effect of BC on the overall adsorption effect is significant.

Figure 10e shows the combined effect of temperature (B) and dosage (D) on *q*_e_ at pH = 7 and *C*_0_ = 40 mg/L. When temperature is between 313 K and 318 K and dosage is 0.04 g/L to 0.06 g/L, *q*_e_ reaches a maximum. As mentioned above, the effect of dosage on the adsorption performance is less significant than other factors. Increasing the temperature alone cannot achieve the optimal adsorption performance of PPy-Fe_3_O_4_/Kaolin. The fact can also be drawn from the very low *p*-value (<0.0001) and relatively low F-value (126.85) of BD in Table S4 and displays that the term of BD is less significant.

Figure 10f displays the interaction of *C*_0_ (C) and dosage of PPy-Fe_3_O_4_/Kaolin (D) on the adsorption capacity *q*_e_ (pH = 7, T = 308 K). The minimal adsorption capacity occurs at the initial concentration and minimal dosage, while the maximum is realized in the ranges of temperature 323–333 K and dosage 0.045–0.06 g/L. As mentioned above, the dosage of PPy-Fe_3_O_4_/Kaolin is an insignificant factor. The F-value and *p*-value of CD are 0.149 and 0.704, respectively, which proves that CD is not a significant factor.

Synthesizing the above analysis, the optimal adsorption conditions for Hg^2+^ ions with PPy-Fe_3_O_4_/Kaolin are pH = 7.2, *T* = 315 K, *C*_0_ = 50 mg/L, dosage = 0.05 g/L, and the adsorption capacity is 317.1 mg/g.

### 3.4. Kinetic Experiments

**Figure 11:**
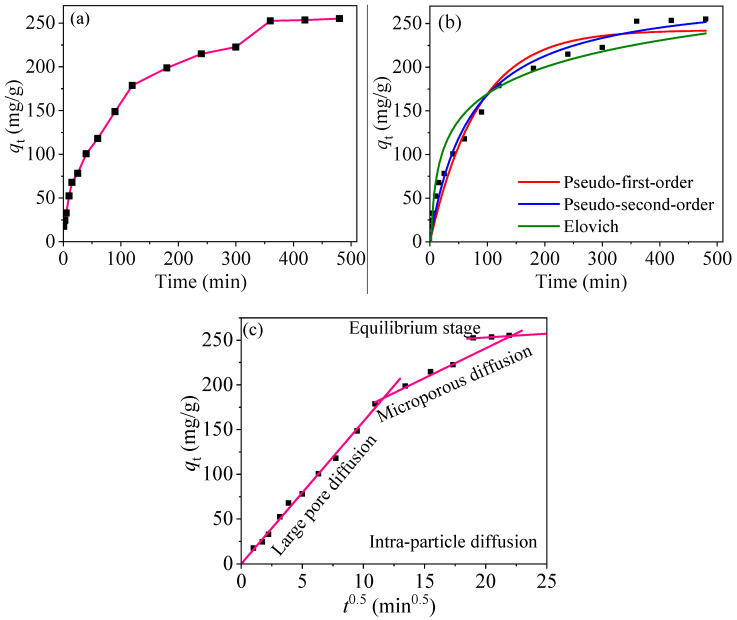
(**a**) Adsorption capacity vs. contact time; Kinetic fitting results: (**b**) and (**c**).

Figure 11a shows the change in adsorption performance over time. According to Figure 11a, the adsorption rate is the fastest within 2 h and the instantaneous adsorption achieves about 178.8 mg/g. In the next four hours, the adsorption rate becomes slow, and finally reaches the adsorption equilibrium with *q*_t_ of 255.2 mg/g after 8 h. Because the surface of PPy-Fe_3_O_4_/Kaolin is rich in amino groups at the beginning of adsorption, the Hg^2+^ ions in water can be quickly adsorbed. Moreover, Hg^2+^ diffuse rapidly from the solution to adsorbent surface due to the concentration gradient of Hg^2+^. The above two facts lead to a quick adsorption. As the amount of effective active sites and concentration gradient of Hg^2+^ ions decrease, adsorption speed decreases slowly and eventually arrives at a saturated state.

Three kinetic models were adopted to deeply study the adsorption performance of PPy-Fe_3_O_4_/Kaolin composites: pseudo-first-order [Equation (S1)], pseudo-second-order [Equation (S2)], and intra-particle diffusion models [Equation (S3)] [40,46]. Additionally, the fitting results are displayed in Figure 11b and Table 2. Table 2 shows that the pseudo-first-order fitting has a low *R*^2^ of 0.966 and the pseudo-second-order fitting have a high *R*^2^ of 0.983 and root mean square error (RMSE) of 11.72, implying a chemical adsorption. Therefore, the pseudo-second-order fitting can be used to describe the adsorption experiment. The fitted *q*_e_ (*q*_e,fit_) of PPy-Fe_3_O_4_/Kaolin in pseudo-second-order equation is 288.2 mg/g, closing to the test data of 255.2 mg/g.

The Elovich model is widely used to fit the chemisorption process. The Elovich model has the fitting results of *R*^2^ = 0.934 and sum of square error (SSE) = 7315, indicating that the adsorption of Hg^2+^ onto PPy-Fe_3_O_4_/Kaolin can be perfectly fitted with the Elovich equation, which proves that the adsorption process is mainly dominated by chemisorption, as shown in the Table 2.

From Figure 11c, the whole adsorption process of Hg^2+^ onto PPy-Fe_3_O_4_/Kaolin can be divided into four phases: (i) boundary layer diffusion [23]. Hg^2+^ ions diffuse from the solution phase to the adjacency of the adsorbent surface (infinitely closing to the surface), and the driving force is derived from the strong concentration differentia of Hg^2+^ ions. The fact can also be concluded from the increasing boundary layer thickness; (ii) large pore adsorption. Corresponded to the fastest adsorption speed, maximal rate constant *K*_d_ of 15.93 min;^−1^, and minimal boundary layer thickness *C*_1_ of 0.163 mg/g [23]; (iii) micropore adsorption. Corresponded to medium adsorption rate constant and *K*_d_ of 6.13 min.^−1^; (iv) equilibrium adsorption. Corresponded to the minimal rate constant and *K*_d_ of 0.882 min.^−1^, and maximal boundary layer thickness *C*_3_ of 235.7 mg/g.

The above results show that the intra-particle diffusion rate decreases as the reaction proceeds, indicating that the third stage (micropore diffusion) and the fourth stage (equilibrium adsorption) are the main adsorption control steps.

### 3.5. Isothermal Experiments

Four isotherm models of the Langmuir [Equation (S4)], Freundlich [Equation (S5)], Temkin [Equation (S6)], and Dubinin–Radushkevich (D-R) [Equation (S7)] were used to fit the adsorption process of PPy-Fe_3_O_4_/Kaolin to study the influence of temperature on the adsorption performance of PPy-Fe_3_O_4_/Kaolin.

The Langmuir isotherm can describe uniform adsorption of monolayers and adsorbates adsorb only one active site, and all active sites are the same [14]. Thus, the affinity of adsorbents to the adsorbents is equal, leading to a constant enthalpy and adsorption activation energy [14].

In the Langmuir model, the separation coefficient *R_L_* [Equation (S8)] means whether the adsorption is favorable. When *R*_L_ > 1, it is not favorable for adsorption, when *R*_L_ < 1, favorable, and when *R*_L_ = 0, irreversible. Freundlich fitting can be employed to research heterogeneous surfaces and the asymmetric distribution of heat and affinity on a heterogeneous surface with different adsorption sites [38,46].

The Temkin isotherm is a kind of real model and it can indicate that the adsorption heat of adsorbates descends linearly with the increase of occupancy. The D-R isotherm can determine the physical and chemical properties of an adsorption process. When the average free energy *E* [Equation (S9)] is less than 8 kJ/mol, representing a physical adsorption, and when 8 kJ/mol < *E* < 16 kJ/mol, it is chemical adsorption.

**Figure 12:**
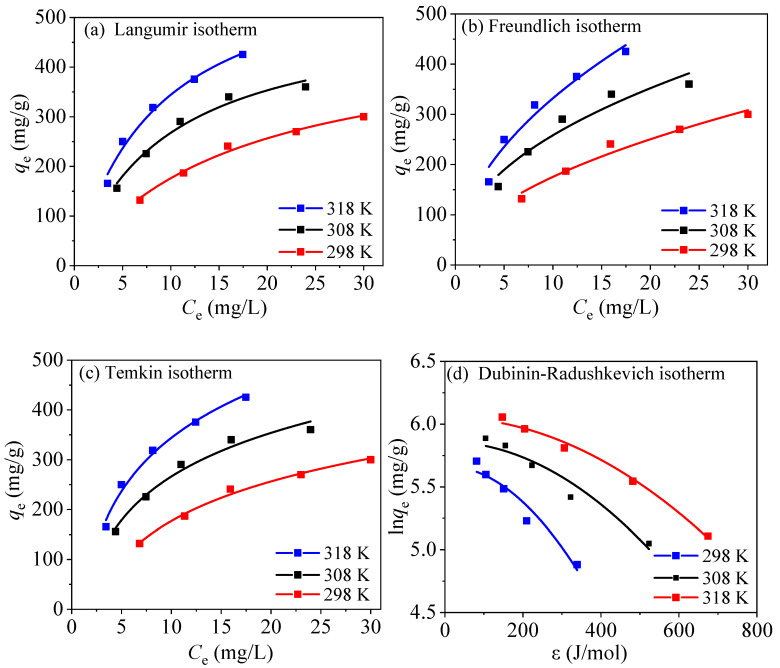
Fitting results of isotherm adsorption with PPy-Fe_3_O_4_/Kaolin.

Figure 12 and Table 3 display the fitting results of four isotherm models with PPy-Fe_3_O_4_/Kaolin. From the fitting results, the correlation coefficient *R*^2^ of the Langmuir fitting is greater than the *R*^2^ of Freundlich fitting (*R*^2^ = 0.969, 0.933, 0.969), which indicated that the absorption process of the adsorbent for mercury can be well represented by the Langmuir model and the adsorption of Hg^2+^ is a single layer adsorption [31,38,40,46]. All of *R*_L_ in the Table 3 are less than 1, indicating that it is favorable for adsorption. The correlation coefficient *R*^2^ of the Temkin model is relatively good, which suggested a very large adsorption potential of the adsorbent for Hg^2+^. In addition, the large value of *b*_T_ also shows that the increase of temperature is beneficial to the interaction between PPy-Fe_3_O_4_/Kaolin and Hg^2+^.

The results of the D-R fitting in Table 3 exhibit that three *E* values (8.3, 12.7, and 15.4 kJ/mol) are located at 8–16 kJ/mol. Obviously, the adsorption of mercury ions onto PPy-Fe_3_O_4_/Kaolin is mainly carried out through a chemical mechanism. The *R*^2^ value of D-R model close to 1, and SSE is small enough, indicating that the D-R model can well describe the adsorption process.

As shown in Table 3, under the experimental conditions, the maximum Langmuir adsorption amount of PPy-Fe_3_O_4_/Kaolin reaches 471.2 mg/g. When compared with other adsorbents (Table S5), although the specific surface area is small, the adsorption performance is high enough.

### 3.6. Recycling and Regeneration Experiment

Recycling and regeneration of materials is also an important criterion for evaluating a material, so reuse experiments were designed. The material after each adsorption of mercury was collected and used in the next adsorption experiment after acid (0.1 M HCl, as regenerant) desorption and repeated several times.

**Figure 13:**
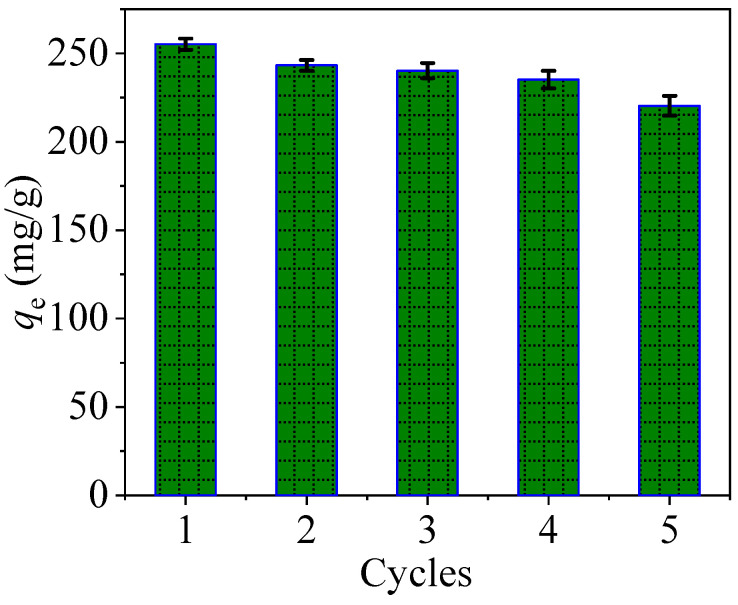
Adsorption performance after repeated use of PPy-Fe_3_O_4_/Kaolin (pH = 7, *C*_0_ = 40 mg/L, *T* = 298 K, *t* = 7 h and dosage of 0.05 g/L).

It can be seen from Figure 13 that the adsorption capacity of PPy-Fe_3_O_4_/Kaolin for mercury is still high after five recycling. When compared with the first time, the capacity only decreases by 13.6%, which indicates that PPy-Fe_3_O_4_/Kaolin has good recyclability.

Combined with the experiments of coexisting ions (Figure S4) and recycling test (Figure 13), the above results suggest that the PPy-Fe_3_O_4_/Kaolin can be a promising adsorbent.

### 3.7. Thermodynamic Experiment

**Figure 14:**
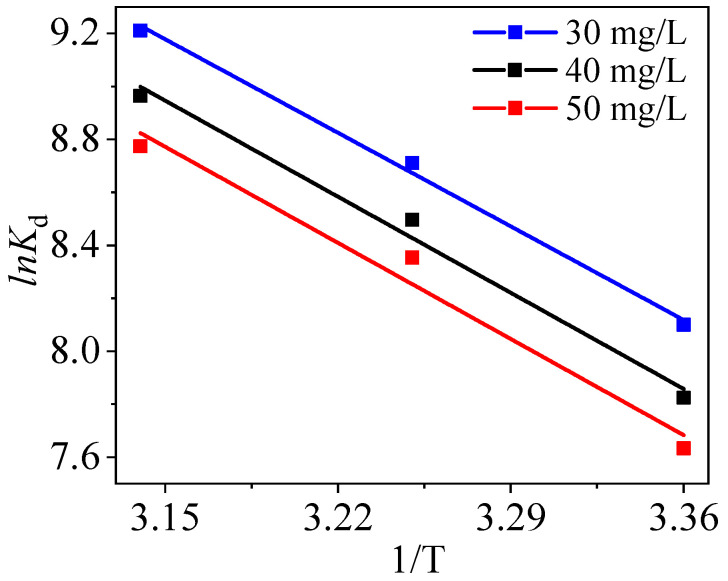
Thermodynamic fitting of mercury adsorption by PPy-Fe_3_O_4_/Kaolin.

The thermodynamic data were linearly fitted in order to investigate the changes in energy and entropy. Gibbs free energy (Δ*G*^0^), enthalpy (Δ*H*^0^), and entropy (Δ*S*^0^) are expressed through Equations (S10) and (S11). The fitting plots and relevant parameters are displayed in Figure 14 and Table 4, respectively.

Three Δ*H*^0^ (41.95, 35.91 and 32.91 kJ/mol) are all positive, representing that the adsorption of Hg^2+^ onto PPy-Fe_3_O_4_/Kaolin is spontaneous and involves chemical reaction, as can be seen from Table 4 [47]. Positive Δ*S*^0^ means that the random degree increases in the composite/solution interface during the process of adsorption [48]. In addition, it can be seen that Δ*G*^0^ gradually descends as ascending temperature, implying that a high temperature is favorable for the adsorption of mercury by PPy-Fe_3_O_4_/Kaolin [27,44]. Additionally, the result is accordant with the data presented in Figure 10a.

### 3.8. Mechanism Speculation

**Figure 15:**
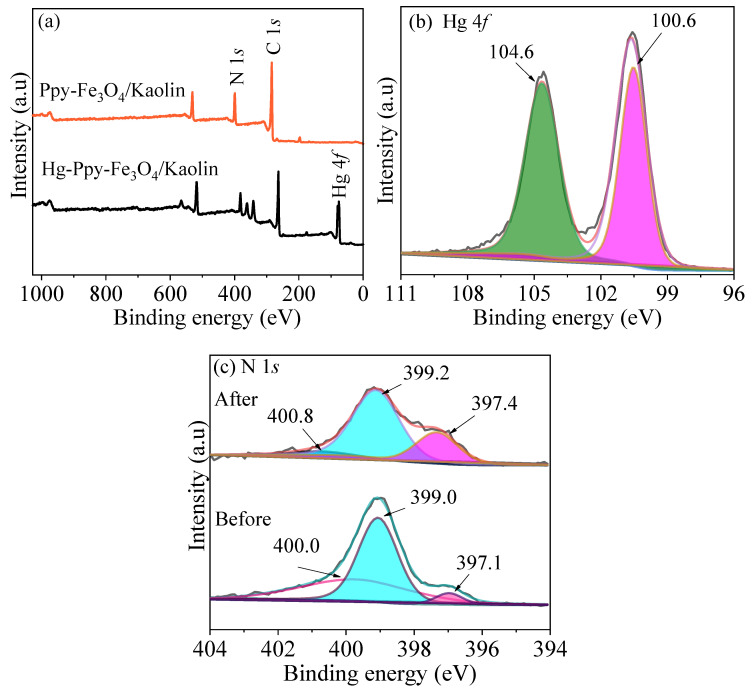
XPS spectra of (**a**) wide spectrum scan, (**b**) Hg 4*f* and (**c**) N 1*s*.

XPS was used to further explore the mechanism of PPy-Fe_3_O_4_/Kaolin adsorption of mercury. From Figure 15a, used PPy-Fe_3_O_4_/Kaolin has a sharp peak at about 100 eV, as compared with the XPS spectrum before adsorption, which can be attributed to Hg 4*f*_5/2_ (104.6 eV) and Hg 4*f*_7/2_ (100.6 eV), as shown in Figure 15b. The result indicates that Hg^2+^ ions are successfully adsorbed onto PPy-Fe_3_O_4_/Kaolin.

Figure 15C is an XPS spectrum of N element before and after adsorption. The N element has three peaks at 397.1 eV, 399.0 eV, and 400.0 eV before adsorption, attributed to -N=, -NH-, and N^+^ [40], respectively. After adsorption, the three peaks of N element are moved to the higher binding energy as a result of the combination of the amino group and Hg^2+^. The change of N in PPy-Fe_3_O_4_/Kaolin was calculated, according to Table 5. The mass percentages of –NH- and N^+^ decreased from 61.8% and 24.7% to 55.1% and 19.1%, respectively, and the mass percentages of -N= increased from 13.5% to 25.8%, which proved that there may be a part of redox during the process of adsorption, and the amino groups participate in the adsorption process.

**Figure 16:**
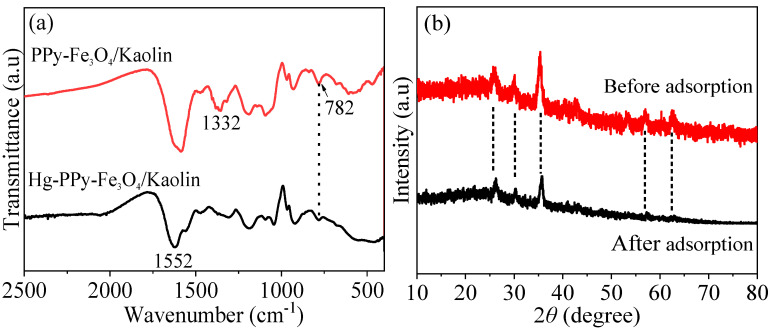

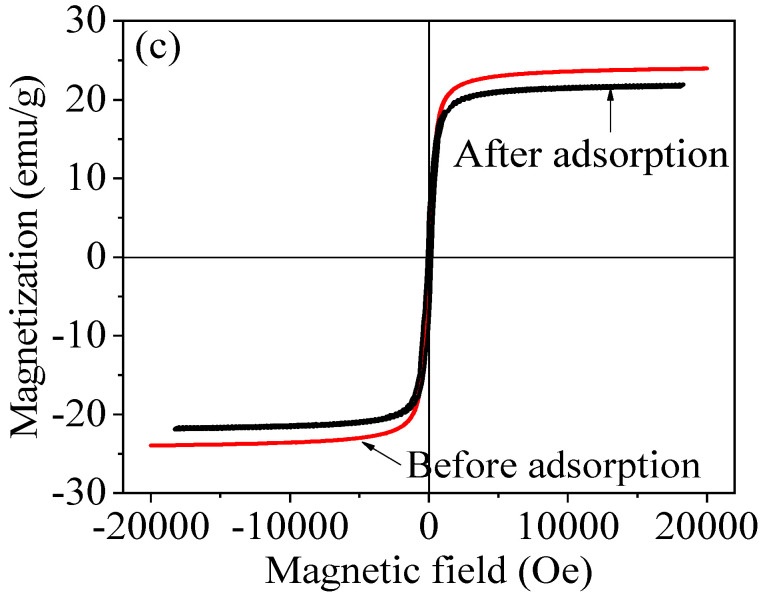
Spectra before and after adsorption with PPy-Fe_3_O_4_/Kaolin: (**a**) FT-IR, (**b**) XRD and (**c**) VSM.

Figure 16a indicates that the peak at 782 cm^−1^ after adsorption is weaker than the FT-IR spectrum of PPy-Fe_3_O_4_/Kaolin before adsorption, which is attributed to the binding of Hg^2+^ ions with the amino group of the PPy-Fe_3_O_4_/Kaolin surface. The combination of the amino group and the mercury ion is a key factor affecting the removal of Hg^2+^ [23].

When compared with the XRD pattern before adsorption, there is no change in the diffraction peak in Figure 16b and no impure peak appears after adsorption. Figure 16c is the hysteresis loop before and after adsorption. It can be seen that after adsorbing mercury ions, PPy-Fe_3_O_4_/Kaolin still has good magnetic properties, which is beneficial for recycling and reducing costs. The above results state clearly that PPy-Fe_3_O_4_/Kaolin has high a crystal and chemical stability when combined with the data of FT-IR, XRD, and VSM patterns revealed in Figure 16.

Generally, mercury has various species in solution, such as Hg^2+^, Hg(OH)^+^, HgCl^+^, and Hg(OH)_2_ [38,40,46]. Additionally, the amount of mercury decreases with the increase of pH until it disappears. When solution pH < 3, the mercury in the solution exists in three species of Hg^2+^ (predominantly), HgOH^+^ (minor), and Hg(OH)_2_ (trace) [38,40,46]. When pH = 4, the amount of Hg(OH)^+^ reaches the maximal value, and, when pH > 6, Hg(OH)_2_ is the main form.

Through the above pH experiment, it can be found that the adsorption performance of PPy-Fe_3_O_4_/Kaolin is greatly affected by solution pH. The higher the solution pH, the better the adsorption of PPy-Fe_3_O_4_/Kaolin for mercury. The reason is that the surface charges of PPy-Fe_3_O_4_/Kaolin are positive at low pH conditions, due to the reaction: -NH_2_ + H^+^ = -NH_3_^+^, as shown in Figure 7. Thus, it is difficult for Hg^2+^ ions to be adsorbed onto PPy-Fe_3_O_4_/Kaolin due to electrostatic repulsion, and Hg^2+^ ions can only be adsorbed by large pore and/or micropore diffusion, as shown in Figure 11c.

As the pH rises, the zeta potential value of PPy-Fe_3_O_4_/Kaolin decreases and the adsorption performance is being improved. The reason is that Hg(OH)^+^, HgCl^+^, and Hg(OH)_2_ have a larger size and faster mobility than Hg^2+^ ions at high pH conditions. These various forms of mercury produce greater electrostatic attraction and faster binding rate with PPy-Fe_3_O_4_/Kaolin, which results in the occurrence of a high adsorption capacity.

**Figure 17:**
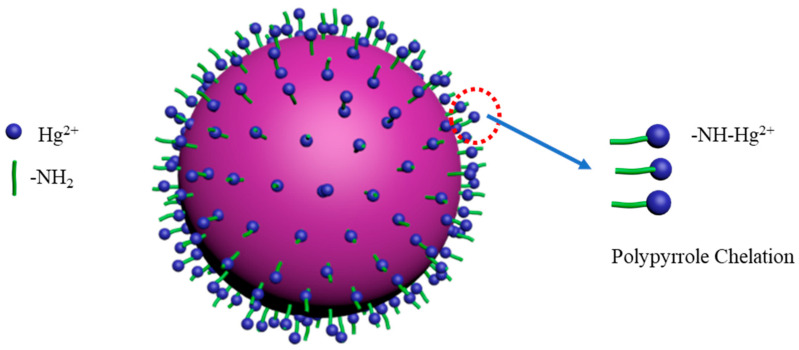
Possible adsorption mechanism of mercury with PPy-Fe_3_O_4_/Kaolin.

In addition, nitrogen atoms in the polypyrrole macromolecular chain are the main active sites for adsorbing mercury ions. At pH > 5, the main specie of mercury is Hg(OH)_2_, which can form a stable metal complex through lone pair electrons on the nitrogen [38,40,46]. Mercury ions can share a lone pair electron with a nitrogen atom in the -N=C- group, as analyzed by the kinetics, isotherm adsorption, and thermo kinetics. Figure 17 displays the possible adsorption mechanism of mercury with PPy-Fe_3_O_4_/Kaolin. However, a pair of lone pairs of electrons on the nitrogen will be slightly protonated under a low pH environment (pH < 5), which hinders the formation of the complex.

Besides, the surface of PPy-Fe_3_O_4_/Kaolin is negatively charged under a condition of a high pH value through the reaction: -NH_2_ + OH^-^ = -NH_2_OH^-^. A high pH value of the solution leads to more negative charges on the surface of PPy-Fe_3_O_4_/Kaolin. These negative charges can react with Hg(OH)^+^, HgCl^+^, and Hg(OH)_2_ by electrostatic action [5], realizing a high adsorption capacity, as shown in Figure 17.

## 4. Conclusions

A kind of polypyrrole functionalized magnetic Kaolin of PPy-Fe_3_O_4_/Kaolin was prepared by a quick, simple, and economical method in order to improve the agglomeration phenomenon and low adsorption capacity of Kaolin. The adsorption performance of PPy-Fe_3_O_4_/Kaolin for Hg^2+^ ions reached 255.2 mg/g at pH = 7. The experimental data and RSM results show that solution pH and temperature are the main effect factors. Additionally, the importance for adsorption performance is pH > *T* > *C*_0_ > dosage. The optimal adsorption conditions for Hg^2+^ ions with PPy-Fe_3_O_4_/Kaolin are pH = 7.2, *T* = 315 K, *C*_0_ = 50 mg/L, dosage = 0.05 g/L, and the adsorption capacity can be 317.1 mg/g. The process of adsorption conforms to the pseudo-second-order and Langmuir models, and it is mainly chemical, spontaneous, and endothermic. The as-prepared PPy-Fe_3_O_4_/Kaolin has excellent reproducibility, dispersity, and chemical stability, and it is easy to be separated from solution via an external magnetic field. The experiments show that PPy-Fe_3_O_4_/Kaolin is an efficient, economical, and safe mercury adsorbent.

## Figures and Tables

**Table 1:** Summary statistics of four modes.

Mode	Std. Dev.	*R* ^2^	*R*_adj_^2^	*R*_pre_^2^	Discrepancy	Press	
Linear	31.09	0.663	0.609	0.543	0.066	76320.67	
2FI	31.95	0.737	0.599	0.546	0.053	75826.07	
Quadratic	0.999	0.999	0.999	0.999	0.001	93.15	Suggested
Cubic	0.652	0.991	0.961	0.884	0.077	299.97	

**Table 2:** Kinetic fitting for adsorption of Hg^2+^ onto PPy-Fe_3_O_4_/Kaolin.

**Pseudo-first-order**															
***q*_e,exp_**	*q*_e,fit_			*k*_1_		*R* ^2^				*x* ^2^		SSE			RMSE
**255.2**	242.5			0.011		0.966				0.963		3871			16.63
**Pseudo-second-order**															
*q*_e,exp_	*q*_e,fit_			*k*_1_		*R* ^2^				*x* ^2^		SSE			RMSE
255.2	288.2			0.004		0.983				0.982		1922			11.72
**Intra-particle diffusion**															
*K*_d-1_	*C*_1_		*R*_1_^2^		*K*_d-2_		*C*_2_		*R*_2_^2^		*K*_d-3_		*C*_3_		*R*_3_^2^
15.93	0.163		0.995		6.13		117.6		0.949		0.882		235.7		0.933
**Elovich**															
*α*		*β*			*R* ^2^			*x* ^2^			SSE			RMSE	
0.01		44.12			0.934			0.921			7315			22.86	

**Table 3:** Adsorption isotherm parameters of PPy-Fe_3_O_4_/Kaolin.

**Langmuir**									
***T* (K)**	***Q*_m_ (mg/g)**		***R*_L_**		***K*_L_ (L/mg)**	***R*^2^**	***x*^2^**	**SSE**	**RMSE**
298	471.2		0.001		28.1	0.991	0.8	173.5	5.9
308	518.7		0.001		55.3	0.981	2.1	556.9	10.6
318	633.8		0.001		75.3	0.986	18.9	5028.5	31.7
**Freundlich**									
*T* (K)		*K*_F_ (L^n^/mg^n−1^/g)			1/n_F_	*R* ^2^	*x* ^2^	SSE	RMSE
298		53.77			0.51	0.969	2.8	553.3	10.5
308		92.16			0.45	0.933	7.3	1903.4	19.5
318		105.72			0.51	0.961	7.1	1653.1	18.2
**Temkin**									
*T* (K)		*b*_T_			*k*_T_	*R* ^2^	*x* ^2^	SSE	RMSE
298		114.5			0.469	0.992	0.7	151.9	5.5
308		126.5			0.821	0.976	2.3	689.5	11.7
318		154.8			0.921	0.991	1.9	423.4	9.2
**Dubinin-Radushkevich**									
*T* (K)		*q*_max_ (mg/g)		*E* (KJ/mol)		*R* ^2^	*x* ^2^	SSE	RMSE
298		381.9		8.3		0.965	0.5	0.02	0.06
308		455.3		12.7		0.963	0.2	0.02	0.06
318		652.7		15.4		0.991	0.1	0.01	0.03

**Table 4:** Thermodynamic parameters of adsorption of mercury by PPy-Fe_3_O_4_/Kaolin.

*C*_0_	Δ*H*^0^	Δ*S*^0^	Δ*G*^0^		
			298 K	308 K	318 K
30	41.95	208.44	−20.08	−22.31	−24.36
40	35.91	187.74	−19.87	−21.96	−23.71
50	32.91	185.51	−19.24	−21.41	−23.03

**Table 5:** Changes in the composition of N element before and after adsorption.

	N^+^ (wt.%)	-NH- (wt.%)	-N= (wt.%)
Before adsorption	24.7%	61.8%	13.5%
After adsorption	19.1%	55.1%	25.8%

## Supplementary Materials

The following are available online at https://www.mdpi.com/2079-4991/10/7/1370/s1, Figure S1: Effect of adsorbent dosage; Figure S2: SEM and EDS micrographs; Figure S3: XRD graph of the materials; Figure S4: Effect of coexisting ions; Table S1: N_2_ adsorption-desorption isothermal data; Table S2: Mass percentage of each element in PPy-Fe_3_O_4_/Kaolin; Table S3: CCD matrix and running results obtained by PPy-Fe_3_O_4_/Kaolin; Table S4: Results of ANOVA of Quadratic mode; Table S5: Comparison of adsorption capacity of mercury by different adsorbents.
