# Supplementary material for: Removal of Hg2+ with Polypyrrole-Functionalized Fe3O4/Kaolin: Synthesis, Performance and Optimization with Response Surface Methodology
Source: Nanomaterials (Basel). 2020 Jul 14;10(7):1370. doi: 10.3390/nano10071370 (PMC7407420; doi:10.3390/nano10071370)
Supplement: Supplementary file 1 [file nanomaterials-10-01370-s001.pdf]

Supporting Information

Removal of Hg^{2+} with Polypyrrole-Functionalized Fe_3O_4/Kaolin: Synthesis, Performance and Optimization with Response Surface Methodology

Zhenfeng Lin [1,2,†], Ziwei Pan [1,†], Yuhao Zhao [1], Lin Qian [1], Jingtao Shen [1], Kai Xia [1], Yongfu Guo [1,3,4,*] and Zan Qu [5]

[1] Center for Separation and Purification Materials &Technologies, Suzhou University of Science and Technology, Suzhou 215011, China; Linzf@sjhb.cn (Z.L.); 1813022033@post.usts.edu.cn (Z.P.); 1713022014@post.usts.edu.cn (Y.Z.); 1911022009@post.usts.edu.cn (L.Q.); 1913022011@post.usts.edu.cn (J.S.); 1713022009@post.usts.edu.cn (K.X.)

[2] Suzhou Sujing Environmental Engineering Co., Ltd., Suzhou 215122, China

[3] Jiangsu Collaborative Innovation Center of Technology and Material of Water Treatment, Suzhou 215009, China

[4] Jiangsu Provincial Key Laboratory of Environmental Science and Engineering, Suzhou University of Science and Technology, Suzhou 215009, China

[5] School of Environmental Science and Engineering, Shanghai Jiao Tong University, Shanghai 200240, China; quzan@sjtu.edu.cn

* Correspondence: yongfuguo@mail.usts.edu.cn; Tel.: +86 512 68092987

† These authors contributed equally to this work.

1. Effect of adsorbent dosage

The preliminary experiments of adsorbent dosage was carried out at pH = 7, C_0 = 40 mg/L and contact time of 12 h. The adsorbent dosage was 0.05, 0.08, 0.1, 0.12 and 0.15 g/L, respectively. The results are shown in the following figure.

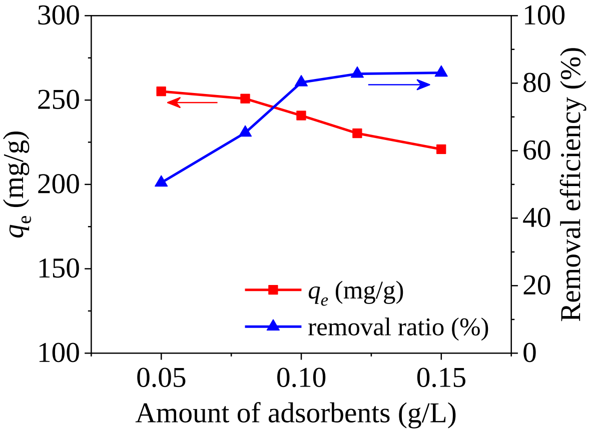

Fig. S1. Effect of adsorbent dosage.

From the above figure, it can be seen that the adsorption capacity of PPy-Fe3O4/Kaolin for mercury was reducing with the increase of addition amount. And the adsorption capacity varied between 220 mg/g and 255 mg/g. The maximum capacity was reached at 0.05 g/L. In addition, it can be known that the removal rate was increased with the increasing dosage. Comprehensively, dosage of 0.05 g/L was selected as the optimal preliminary parameter in the subsequent studies.

2. Sample characterizations

The values of surface area (BET) were decided by N_2 adsorption-desorption instrument (Micromeritic TriStarII 3020, Norcross, GA, USA). The morphology was observed by scanning electron microscope (SEM, FEI, Phenom, Hillsboro, TX, USA) and transmission electron microscopy (TEM, JEM-2100F, Tokyo, Japan). X-ray Diffraction analysis (XRD, Bruker D8 Advance Bruker, Karlsruhe, Germany) was applied to investigate the crystallization and phase. Functional groups were identified by Fourier transform infrared spectrophotometer (FT-IR, Thermo, Nicolet-6700, Thermo Scientific, Waltham, MA, USA). Magnetic strength was compared by vibrating sample magnetometer (VSM, Quantum design, PPMS-9, Quantum Design, San Diego, CA, USA). Elements compositions were confirmed by energy-dispersive spectrometer (EDS) and X-ray photoelectron spectroscopy (XPS, Thermo Scientific, 250Xi, Thermo Scientific, Waltham, MA, USA). The concentration of Hg^{2+} ions at any time t (min) was quantified using ICP-OES. The solution of ultrasonic dispersion used an ultrasonicator (SB-5200DT, SCIENTZ, China).

3. SEM-mapping

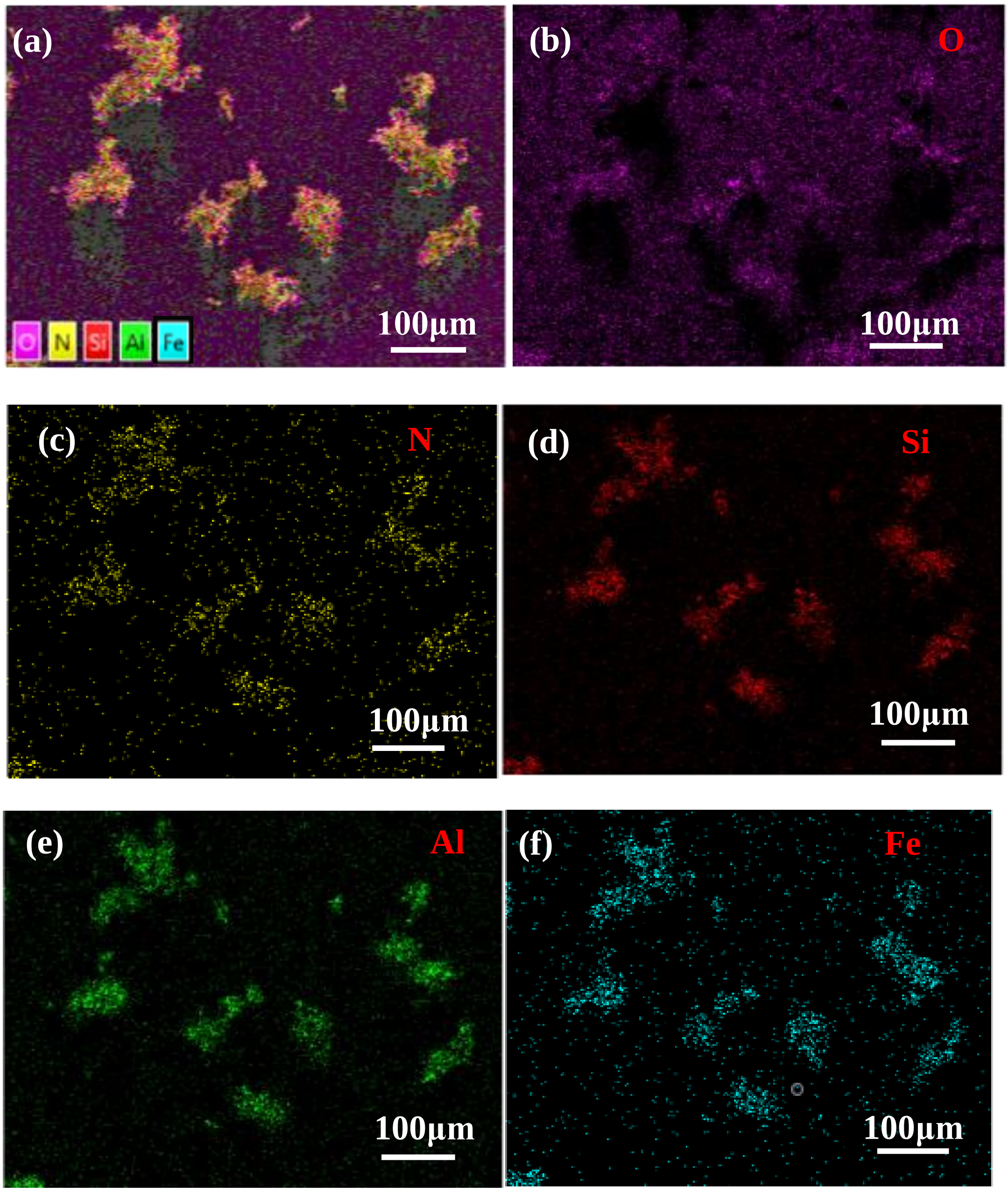

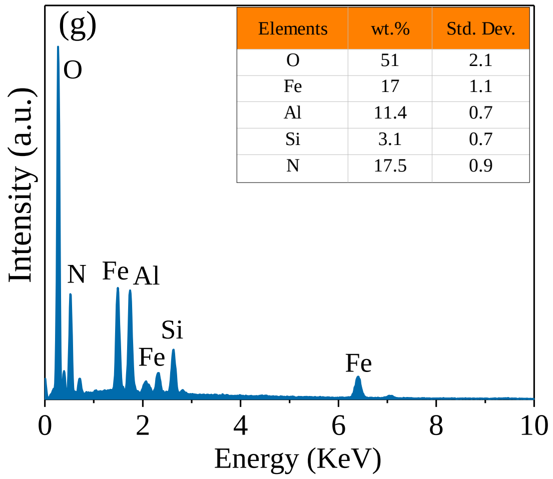

Fig. S2. (**a**) SEM micrograph with X-ray elemental area scanning, (**b**) EDS mapping of PPy-Fe_3O_4/Kaolin (**c**) O, (**d**) N, (**e**) Si, (**f**) Al and (g) Fe; (**g**) distribution of elements.

Table 1. N_2 adsorption-desorption isothermal data of samples.

Samples	BET (m^2/g)	Total pore volume (cm^3/g)	Pore diameter (nm)
Kaolin	10.30	0.03	11.53
Fe_3O_4/Kaolin	39.92	0.07	6.65
PPy-Fe_3O_4/Kaolin	84.19	0.17	8.31

4. XRD

XRD spectra of Kaolin, Fe_3O_4/Kaolin and PPy-Fe_3O_4/Kaolin are shown in **Fig. S3**. It can be seen from the XRD spectrum that the diffraction peaks of kaolin (2θ = 26.69°, 34.98°, 35.40°, 35.96°, 36.68°, 37.70°, 68.42°, 39.96°, 54.98° and 62.23°) show the crystallinity of kaolin [1]. In the XRD spectrum of Fe_3O_4/Kaolin, the peaks at 2θ of 30.50°, 35.42°, 43.22°, 53.62°, 57.12° and 62.63° can be ascribed to (220), (311), (400), (422), (511) and (440) of Fe_3O_4 (JCPDS No. 19-0629) [2]. Compared with Fe_3O_4/Kaolin, the spectrum of PPy-Fe_3O_4/Kaolin has a broad peak between 15° and 30°, which may be caused by the typical characters of the amorphous polymers [3]. Due to the coating of polypyrrole, the characteristic peak of Fe_3O_4/Kaolin is weakened. The above results suggest that polypyrrole is successfully synthesized onto Fe_3O_4/Kaolin.

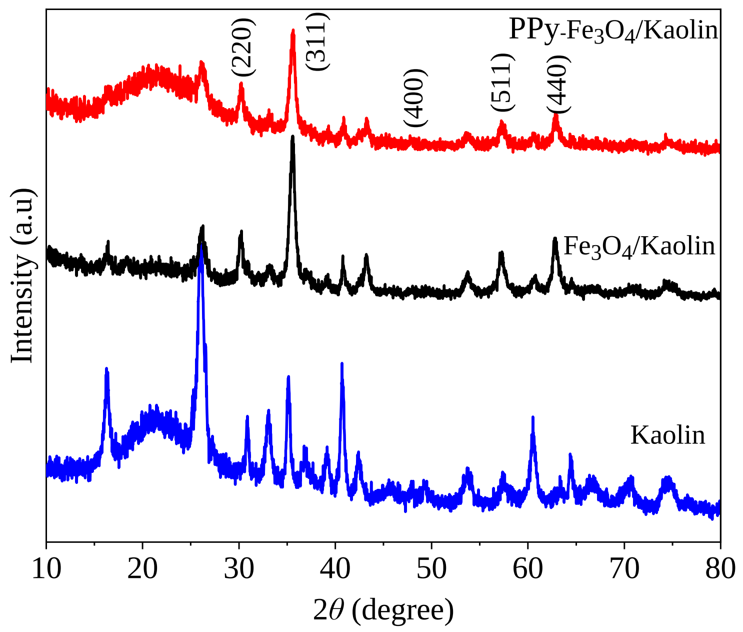

Fig. S3. XRD of Kaolin, Fe_3O_4/Kaolin, PPy-Fe_3O_4/Kaolin.

Table S2. Mass percentage of each element in PPy-Fe_3O_4/Kaolin.

Name	Start B.E.	Peak B.E.	End B.E.	Wt. (%)
C 1s	297.98	284.06	279.18	63.9
O 1s	542.18	530.81	525.08	12.8
Si 2p	107.38	102.62	96.08	1.3
Al 2p	84.98	80.87	65.18	2.5
N 1s	406.98	399.07	392.68	16.9
Fe 2p	739.98	710.4	700.18	2.6

Table S3. CCD matrix and running results obtained by PPy-Fe_3O_4/Kaolin.

Std.	Run	Variables				Response
		pH (A)	*T* (B)	C_0 (C)	Dosage (D)	(q_e)
16	1	8	40	50	0.06	301
5	2	6	30	50	0.04	185
2	3	8	30	30	0.04	225
19	4	7	25	40	0.05	245
20	5	7	45	40	0.05	318
22	6	7	35	60	0.05	361
26	7	7	35	40	0.05	287
14	8	8	30	50	0.06	284
7	9	6	40	50	0.04	186
18	10	9	35	40	0.05	274
21	11	7	35	20	0.05	215
24	12	7	35	40	0.07	251
13	13	6	30	50	0.06	217
27	14	7	35	40	0.05	252
9	15	6	30	30	0.06	208
10	16	8	30	30	0.06	235
28	17	7	35	40	0.05	287
8	18	8	40	50	0.04	316
29	19	7	35	40	0.05	281
1	20	6	30	30	0.04	175
17	21	5	35	40	0.05	112
4	22	8	40	30	0.04	283
6	23	8	30	50	0.04	282
12	24	8	40	30	0.06	268
30	25	7	35	40	0.05	251
11	26	6	40	30	0.06	203
25	27	7	35	40	0.05	253
3	28	6	40	30	0.04	193
15	29	6	40	50	0.06	197
23	30	7	35	40	0.03	231

Table S4. Results of ANOVA of Quadratic mode.

Terms	Sum of square	df	Mean square	F-values	p-values	
Mode	69260.83	14	11626.84	4981	< 0.0001	Significant
A-(pH)	36955.19	1	93001.5	37209	< 0.0001	
B-(T)	1783.82	1	7490.67	1796	< 0.0001	
C-(C0)	2972.64	1	6800.67	1563	< 0.0001	
D-(Dosage)	507.40	1	3360.67	510	< 0.0001	
AB	1438.95	1	3721	1448	< 0.0001	
AC	1712.47	1	4692.25	633	< 0.0001	
AD	629.01	1	1936	252	< 0.0001	
BC	308.21	1	702.25	172.49	< 0.0001	
BD	509.50	1	1369	126.85	< 0.0001	
CD	2.46	1	42.25	0.149	0.704	Insignificant
A^2	15493.46	1	32725.76	1560	< 0.0001	
B^2	1145.90	1	6309.33	1153	< 0.0001	
C^2	4499.61	1	8928.05	4530	< 0.0001	
D^2	3885.31	1	1943.05	3912	< 0.0001	
Residual	17.88	18	0.99			
Lack of Fit	2674.08	10	1.57	5.75	0.069	Insignificant
Pure Error	2.18	8	0.27			
Cor Total	69278.72	32				

5. Models of adsorption kinetics

Three kinetic models were adopted to fit the experimental data: pseudo-first-order model [Eq. (S1)], pseudo-second-order model [Eq. (S2)] and intra-particle diffusion model [Eq. (S3)].

$$q_t = (1 - \frac{1}{e^{k_1 t}}) q_e \quad \text{(S1)}$$

$$q_t = \frac{k_2 t q_e^2}{1 + k_2 t q_e} \quad \text{(S2)}$$

$$q_t = k_{d-i} t^{\frac{1}{2}} + C_i \quad \text{(S3)}$$

where, q_t (mg/g) represents instantaneous adsorption capacity; k_1 (min^{-1}), k_2 (g/mg/min) and k_{d-i} (min^{-1}) are all rate constants; C_i (mg/g) is the boundary layer thickness.

Table S5. Comparison of adsorption capacity of mercury by different adsorbents.

Adsorbents	BET (m^2/g)	pH	Fitting models	Q_m (mg/g)	Ref.
MGO-PAMAM-G3.0	40.93	3	Langmuir	113.71	[4]
Magnetic Fe_3O_4 GO	58.6	6	Langmuir	71.3	[5]
$CoFe_2O_4$–rGO	69.9	4.6	Langmuir	157.9	[6]
Coal based activated carbon	442.3	4	Langmuir	48.9	[7]
$CoFe_2O_4$@SiO_2–NH_2	17.08	7	Langmuir	149.3	[8]
Modified nanoporous	1198.4	6	Langmuir	8.9	[9]
N-donor arranged SBA15	715.4	2.5	Langmuir	8.8	[10]
Polypyrrole/SBA-15	97.6	8	Langmuir	200	[11]
PPy/SH-Beta/MCM-41	-	8	Freundlich	157.4	[12]
Magnetic CNTs/Fe_3O_4	97.163	6.5	Langmuir	65.52	[13]
MBT-GO	-	6.9	Langmuir	107.52	[14]
Diatom silica-SH	19.3	6	Langmuir	131.7	[15]
SBA-15-SH	50.94	8	Freundlich	195.6	[16]

Adsorbents	BET (m^2/g)	pH	Fitting models	Q_m (mg/g)	Ref.
PPy-Fe_3O_4/Kaolin	84.19	7	Langmuir	471.2	This work

6. Adsorption isotherm models

$$q_e = \frac{Q_m k_L C_e}{1 + k_L C_e} \quad \text{(S4)}$$

$$q_e = k_F C_e^{\frac{1}{nF}} \quad \text{(S5)}$$

$$q_e = \frac{RT}{b_t} \text{In}(K_T C_e) \quad \text{(S6)}$$

$$q_\text{e} = q_\text{max} e^{(-\beta\varepsilon^2)} \quad \text{(S7)}$$

$$R_L = \frac{1}{1 + K_L C_0} \quad \text{(S8)}$$

$$E = \frac{1}{(2\beta)^{\frac{1}{2}}} \quad \text{(S9)}$$

where, Q_m (mg/g) is the maximal single layer adsorption capacity. K_L (L/mg), K_F, q_{max} (mg/g) and K_T (L/mg) are Langmuir, Freundlich, Temkin and Dubinin-Radushkevich constants. 1/n_F is the uneven factor. T (K) represents thermodynamic temperature. R is gas constant (8.314 J/mol/K). β is activity coefficient associated with adsorption energy. R_L is the separation constant of the Langmuir isotherm model. E (KJ/mol) is the average adsorption free energy.

7. Effect of coexisting ions

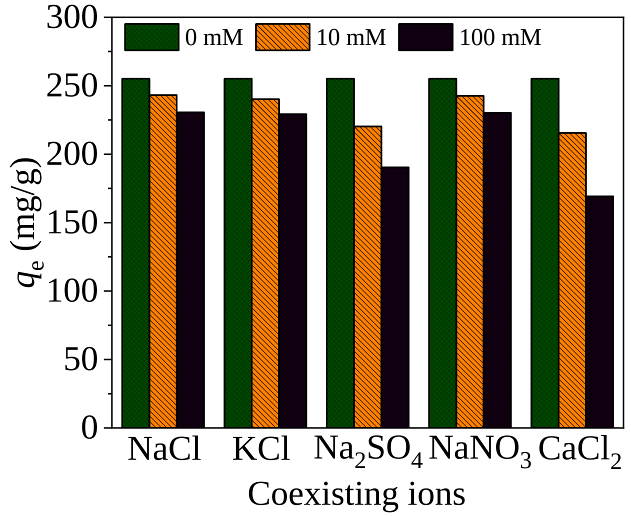

Fig. S4. Effect of coexisting ions (pH = 7, C_0 = 40 mg/L, T = 273 K, *t* = 420 min and dosage of 0.05 g/L).

In this experiment, six common types of ions were selected to evaluate the effect of coexisting ions on the adsorption of mercury ions by PPy-Fe_3O_4/Kaolin, which are Na^+, K^+, Ca^{2+}, Cl^-, NO_3^-, and SO_4^{2-}, respectively. The above six ions are separately added to the mercury solutions whose concentrations at 40 mg/L and pH = 7. Subsequently, 0.05 g/L of adsorbent was added to the solution, and the solution was shaken for 7 h at 298 K.

Nature water often contains different ions, which may affect the adsorption of mercury ions by PPy-Fe_3O_4/Kaolin. Thus, three cations (Na^+, K^+, Ca^{2+}) and three anions (Cl^-, NO_3^-, SO_4^{2-}) were used to study the effect of ions on the adsorption effect.

As can be seen from **Fig. S4**, with the increasing ion concentration, the ability of PPy-Fe_3O_4/Kaolin to adsorb mercury decreases. Among the three anions, SO_4^{2-} has the greatest influence on the adsorption performance of PPy-Fe_3O_4/Kaolin. When the concentration is 10 mM and 100 mM, the adsorption capacity of PPy-Fe_3O_4/Kaolin for mercury is reduced by 13.7% and 25.4%, respectively, compared with the concentration of 0 mM.

Among the three cations, Ca^{2+} has the greatest influence on the adsorption performance. At a concentration of 10 mM and 100 mM, the adsorption capacity for mercury is reduced by 15.6% and 33.7%, respectively. This may be because Ca^{2+} is bivalent and can occupy two active adsorption sites.

8. Model of thermodynamics

$$\Delta G^0 = - RT \ln K_d \tag{S10}$$

$$\ln K_d = \frac{\Delta S^0}{R} - \frac{\Delta H^0}{RT} \tag{S11}$$

where, K_d is a constant and can be calculated from q_e/C_e; ΔH^0 and ΔS^0 are the slope and intercept of the lnK_d *vs*. 1/T, respectively.

Reference

[1] A. Özcan, A. Atılır Özcan, Y. Demirci, E. Şener, Preparation of Fe_2O_3 modified kaolin and application in

heterogeneous electro-catalytic oxidation of enoxacin, Appl. Catal. B. 200 (2017) 361-371.
[2] K. Xia, Y. Guo, Q. Shao, Q. Zan, R. Bai, Removal of mercury (II) by EDTA-functionalized magnetic $CoFe_2O_4@SiO_2$ nanomaterial with core-shell structure, Nanomaterials 9 (2019) 1532-1554.
[3] Y. Liao, X. Wang, W. Qian, Y. Li, X. Li, D.G. Yu, Bulk synthesis, optimization, and characterization of highly dispersible polypyrrole nanoparticles toward protein separation using nanocomposite membranes, J. Colloid Interface Sci. 386 (2012) 148-157.
[4] Y. Ma, D. Xing, W. Shao, X. Du, P. La, Preparation of polyamidoamine dendrimers functionalized magnetic graphene oxide for the adsorption of Hg(II) in aqueous solution, J. Colloid Interface Sci. 505 (2017) 352-363.
[5] Y. Guo, J. Deng, J. Zhu, X. Zhou, R. Bai, Removal of mercury (II) and methylene blue from a wastewater environment with magnetic graphene oxide: adsorption kinetics, isotherms and mechanism, RSC Adv. 6 (2016) 82523-82536.
[6] Y. Zhang, L. Yan, W. Xu, X. Guo, L. Cui, L. Gao, Q. Wei, B. Du, Adsorption of Pb(II) and Hg(II) from aqueous solution using magnetic $CoFe_2O_4$-reduced graphene oxide, J. Mol. Liq. 191 (2014) 177-182.
[7] Y. Guo, Z. Wang, X. Zhou, R. Bai, Removal of mercury (II) from aqueous solution with three commercial raw activated carbons, Res. Chem. Intermed. 43 (2016) 2273-2297.
[8] X. Wang, Z. Zhang, Y. Zhao, K. Xia, Y. Guo, Z. Qu, R. Bai, A mild and facile synthesis of amino functionalized $CoFe_2O_4@SiO_2$ for Hg(II) removal, Nanomaterials 8 (2018) 673-693.
[9] M. Anbia, S. Amirmahmoodi, Removal of Hg(II) and Mn(II) from aqueous solution using nanoporous carbon impregnated with surfactants, Arabian J. Chem. 9 (2016) S319-S325.
[10] M.H. Dindar, M.R. Yaftian, M. Hajihasani, S. Rostamnia, Refinement of contaminated water by Cr(VI), As(V) and Hg(II) using N-donor ligands arranged on SBA-15 platform; batch and fixed-bed column methods, J. Taiwan Inst. Chem. Eng. 67 (2016) 325-337.
[11] M. Shafiabadi, A. Dashti, H.A. Tayebi, Removal of Hg(II) from aqueous solution using polypyrrole/SBA-15 nanocomposite: Experimental and modeling, Synth. Met. 212 (2016) 154-160.
[12] H. Javadian, M. Taghavi, Application of novel olypyrrole/thiolfunctionalized zeolite Beta/MCM-41 type mesoporous silica nanocomposite for adsorption of Hg^{2+} from aqueous solution and industrial wastewater: Kinetic, isotherm and thermodynamic studies, Appl. Surf. Sci. 289 (2014) 487-494.
[13] C. Zhang, J. Sui, J. Li, Y. Tang, W. Cai, Efficient removal of heavy metal ions by thiol-functionalized superparamagnetic carbon nanotubes, Chem. Eng. J. 210 (2012) 45-52.
[14] A.S. Krishna Kumar, S.J. Jiang, W.L. Tseng, Facile synthesis and characterization of thiol-functionalized graphene oxide as effective adsorbent for Hg(II), J. Environ. Chem. Eng. 4 (2016) 2052-2065.
[15] Y. Yu, J. Addai-Mensah, D. Losic, Functionalized diatom silica microparticles for removal of mercury ions, Sci. Technol. Adv. Mater. 13 (2012) 015008.
[16] Y. Shen, N. Jiang, S. Liu, C. Zheng, X. Wang, T. Huang, Y. Guo, R. Bai, Thiol functionalization of short channel SBA-15 through a safe, mild and facile method and application for the removal of mercury (II), J. Environ. Chem. Eng. 6 (2018) 5420-5433.
